# S4D-ECG: A Shallow State-of-the-Art Model for Cardiac Abnormality Classification

**DOI:** 10.1007/s13239-024-00716-3

**Published:** 2024-02-08

**Authors:** Zhaojing Huang, Luis Fernando Herbozo Contreras, Leping Yu, Nhan Duy Truong, Armin Nikpour, Omid Kavehei

**Affiliations:** 1 School of Biomedical Engineering, The University of Sydney, Darlington, NSW Australia; 2 Central Clinical School of the University of Sydney, Camperdown, NSW Australia; 3 Royal Prince Alfred Hospital, Camperdown, NSW Australia

**Keywords:** ECG analysis, S4D model, Noise robustness, Classification accuracy, Low complexity architecture

## Abstract

**Purpose:**

This study introduces an algorithm specifically designed for processing unprocessed 12-lead electrocardiogram (ECG) data, with the primary aim of detecting cardiac abnormalities.

**Methods:**

The proposed model integrates Diagonal State Space Sequence (S4D) model into its architecture, leveraging its effectiveness in capturing dynamics within time-series data. The S4D model is designed with stacked S4D layers for processing raw input data and a simplified decoder using a dense layer for predicting abnormality types. Experimental optimization determines the optimal number of S4D layers, striking a balance between computational efficiency and predictive performance. This comprehensive approach ensures the model's suitability for real-time processing on hardware devices with limited capabilities, offering a streamlined yet effective solution for heart monitoring.

**Results:**

Among the notable features of this algorithm is its strong resilience to noise, enabling the algorithm to achieve an average F1-score of 81.2% and an AUROC of 95.5% in generalization. The model underwent testing specifically on the lead II ECG signal, exhibiting consistent performance with an F1-score of 79.5% and an AUROC of 95.7%.

**Conclusion:**

It is characterized by the elimination of pre-processing features and the availability of a low-complexity architecture that makes it suitable for implementation on numerous computing devices because it is easily implementable. Consequently, this algorithm exhibits considerable potential for practical applications in analyzing real-world ECG data. This model can be placed on the cloud for diagnosis. The model was also tested on lead II of the ECG alone and has demonstrated promising results, supporting its potential for on-device application.

## Introduction

The electrocardiogram (ECG) is used as a wearable and invasive diagnostic modality for monitoring the heart’s electrical activity. This diagnostic technique entails strategically placing electrodes on the patient’s body to capture the ECG signal. The widely recognized and standardized method for electrode placement in ECG monitoring is the 12-lead ECG, which incorporates chest and limb leads. These leads enable the measurement of heart electrical activities along horizontal and vertical planes [1]. Notably, in the study conducted by [2], the convolution neural network (CNN) is observed that their model achieves a slightly higher level of performance in comparison to our model. However, their approach employs a significantly more complex architecture, utilizing a 15-layer CNN. In comparison, our model demonstrates competitive results with a more streamlined and easier-to-explain architecture. This underscores the efficiency and effectiveness of our model in achieving comparable outcomes with a reduced computational burden. The proposed model exhibits versatile applicability, with the capability to be seamlessly integrated into cloud-based systems, facilitating its utilization by medical professionals for diagnosing cardiac abnormalities. Furthermore, its adaptability to operate effectively under single-electrode conditions opens up the possibility of embedding it directly into medical devices for on-site diagnosis and monitoring, thereby enhancing its accessibility and potential impact in clinical settings.

### Background

The structured state space for sequence modeling (S4) architecture is a state space model that was introduced as an innovative approach for tasks involving extremely long-range sequences in many fields, such as audio processing and natural language, and has proven its effectiveness in handling dependencies in sequences that extend across tens of thousands of steps [3]. They have shown to be very effective and demonstrated promising alternatives to sequence models such as CNNs or recurrent neural networks (RNNs) [4]. Several prior works investigated the effectiveness of S4 models for ECG analysis. Notably, [5] developed a S4 model that achieved a supervised performance of $0.94\pm 0.0016$ when tested with the PTB-XL dataset [6]. This significant achievement demonstrates the effectiveness and potential of S4 models in accurately analyzing and classifying ECG signals. In addition, [7] developed an S4 model as a diffusion-based approach for generating ECG data. Their model conditioned a diverse set of 71 ECG statements in a multi-label setting, representing an unprecedented degree of complexity [7]. The work by [7] showcases the versatility of S4 models and their ability to handle complex ECG data generation tasks.

Building on the success of S4, [4] introduced a diagonal state space sequence (S4D) algorithm as a simple diagonal version of S4 with highly simplified coding and comparable performance to S4 on many different time-series data modalities. In our current study, we utilize the S4D algorithm to demonstrate its *out-of-sample generalization*, *robustness against noise*, and *efficacy of performance without pre-processing*. We tested, achieved and reported these objectives on hospital-grade 12-lead electrocardiogram (ECG) data. To highlight the overhead of pre-processing, it is needed to highlight that pre-processing of 14,332 records of 12-lead ECG datasets on an Intel Xeon CPU E5-2630 v3 (at 2.4 GHz) demands about 3 min of computational time. The model efficiently conserves computational resources by eliminating the need for pre-processing the data. Our current study primarily focuses on 12-lead ECG data, which is commonly used in clinical settings such as intensive care units (ICUs) or comprehensive cardiac monitoring centers. As S4D promises hugely reduced computational overhead, we also demonstrate its performance on single-lead ECG to demonstrate its potential use in injectable cardiac monitor (ICM) such as Medtronic’s LINQ^TM^ and Abbott’s Confirm Rx^TM^ and the artificial applications in single-lead ECG processing [8, 9]. In the following parts of this paper, we discuss S4 and S4D models in further detail.

**Fig. 1:**
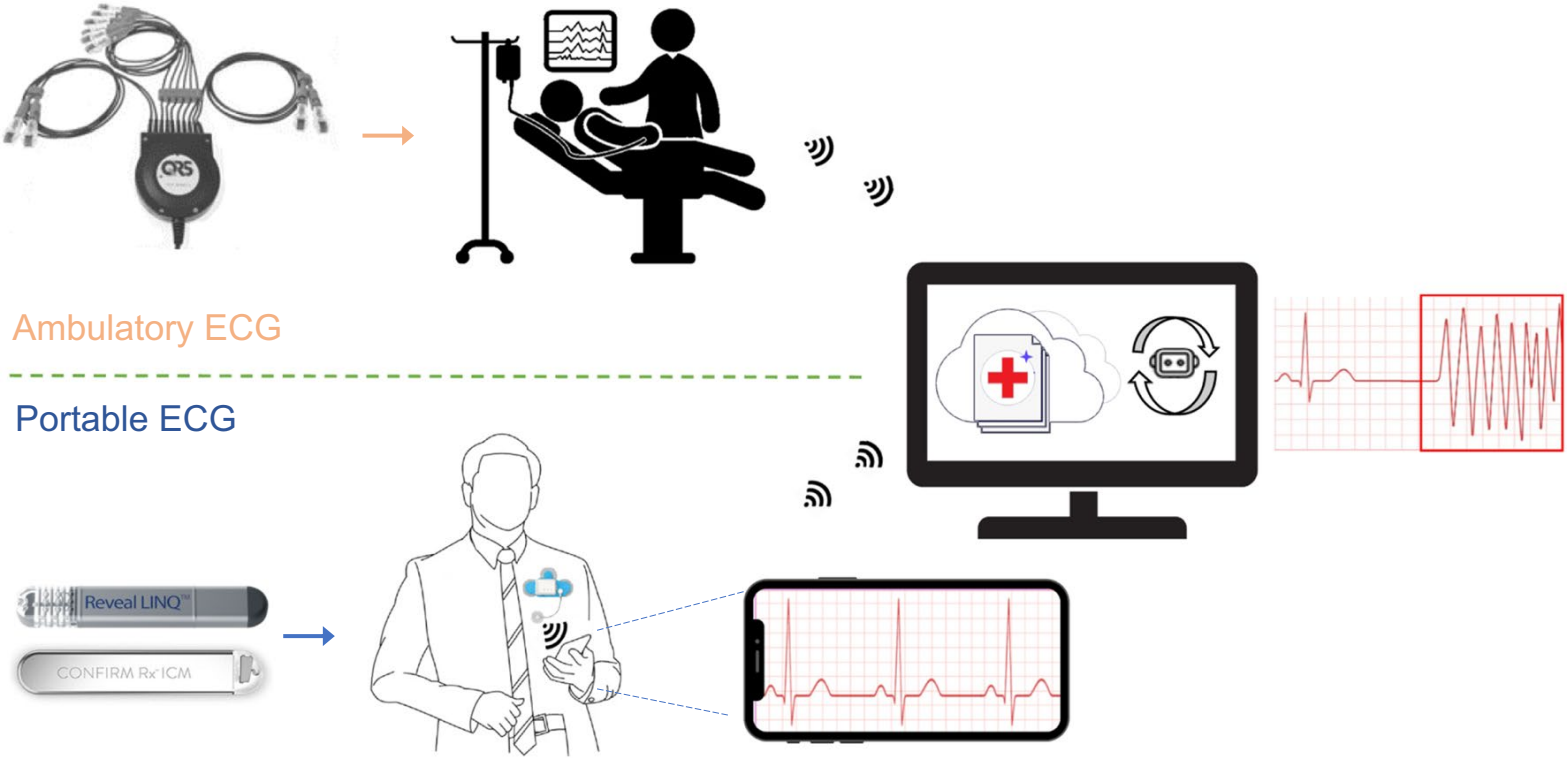
The proposed AI-based abnormality detection model can be implemented in hospital settings and portable devices for assistance with the detection of abnormal heart rhythms. For example, Universal ECG and VectraplexECG^TM^ System (by VectraCor) (top [10]) and Medtronic’s LINQ^TM^ or Abbott’s Confirm Rx^TM^ ICMs devices (bottom [8])

Figure 1 proposes a visionary device for early detection and timely intervention of abnormalities. Healthcare professionals can benefit from this technology by focusing on specific points where the abnormality is detected, reducing their workload. Integrating AI in portable devices also enables remote monitoring and telemedicine, which is particularly beneficial for individuals in remote areas.

## Prerequisite

### Structured State Space Sequence (S4) Model

The structured state space for sequence modeling (S4) is a deep learning model that processes long data sequences as a compelling alternative to more traditional sequence models such as RNNs [3]. It has been shown recognizing long-range dependencies in sequential and time-series data, is critical when dealing with complex data sets exhibiting intricate patterns and relationships. The continuous-time state-space model converts a one-dimensional input signal *u*(*t*) into a latent state *x*(*t*) of higher dimensions before projecting it onto a one-dimensional output signal *y*(*t*).

1 $$\begin{aligned} \dot{x}(t)= & {} Ax(t) + Bu(t) \end{aligned}$$

2 $$\begin{aligned} y(t){} & {} = Cx(t) + Du(t) \end{aligned}$$

Given matrices *A*, *B*, *C*, and *D* of appropriate sizes, the state-space model can be discretized using various techniques to model sequences with a fixed step size $\Delta$. One commonly used technique is the zero-order hold method. By employing this method, we can derive a simple linear recurrence relationship as the following

3 $$\begin{aligned} {x}_{n}= & {} \bar{A}{x}_{n-1} + \bar{B}{u}_{n}, \end{aligned}$$

4 $$\begin{aligned} {y}_{n}= & {} \bar{C}{x}_{n} + \bar{D}{u}_{n}, \end{aligned}$$

here, $\bar{A}$, $\bar{B}$, $\bar{C}$, and $\bar{D}$ can be calculated as functions of *A*, *B*, *C*, *D*, and $\Delta$.

### Diagonal State Space Sequence (S4D) Model

Using a structured state space model formulation, the S4 model is designed to capture the structure and dependencies in time-series data. It incorporates a parameterization technique known as the diagonal plus low-rank (DPLR) matrix to represent the state matrix, allowing it to capture hierarchical dependencies efficiently [4]. On the other hand, the S4D model simplifies the parameterization by using a diagonal state space model formulation [4]. This means the state matrix is a diagonal matrix without the low-rank term. This simplification reduces the computational complexity and makes the model easier to implement and understand. By removing the low-rank term from the parameterization, the S4D model achieves a simpler mathematical representation than the S4 model.

## Datasets

The proposed model in this study was evaluated using two different datasets: the 12-lead ECG dataset and the Telehealth Network of Minas Gerais, Brazil (TNMG) dataset [11]. The 12-lead ECG dataset, also known as the CPSC dataset, was created as part of the China Physiological Signal Challenge 2018 [12]. This dataset is designed to identify rhythm and morphology abnormalities in 12-lead ECGs automatically. It serves as the primary dataset for training the model in this study. The TNMG dataset was used as a separate test dataset to assess the model’s generalisation performance. The TNMG dataset is a collection of ECG data from the Telehealth Network of Minas Gerais project, which focuses on remote healthcare delivery in the Minas Gerais region of Brazil. This dataset provides an opportunity to evaluate how well the model performs on real-world data outside the CPSC dataset. By training the model on the CPSC dataset and testing it on the TNMG dataset, the researchers aimed to assess its ability to generalize and perform well on unseen data. This evaluation helps determine the model’s effectiveness and reliability in real-world scenarios.

### CPSC

The CPSC dataset has been compiled to encompass 12-lead electrocardiograms (ECGs) recorded at a sampling rate of 500 Hz. The dataset is notable for collecting ECGs from 6877 patients exhibiting various cardiovascular conditions and common rhythms, all of which have been expertly labeled [12]. To assess the model’s performance effectively, we trained it using eight distinct abnormalities from the dataset. Table 1 provides further elaboration on the eight selected abnormalities.

**Table 1:** Classifications of abnormalities in CPSC dataset

Type	Acronyms	Description
First degree atrioventricular block	1dAVb	The presence of a PR interval exceeding 0.2 s serves as an indication [13]
Atrial fibrillation	AF	The most frequently observed form of abnormality is identified by a fast and irregular heartbeat [14]
Left bundle branch block	LBBB	The activation sequence in the right ventricle preceding the left ventricle leads to alterations in the workload, mechanics, and perfusion of the left ventricle [15]
Right bundle branch block	RBBB	When there is a disruption in signal transmission within the His–Purkinje system, it can result in delayed depolarization in the right ventricle. This condition can then affect the electrical activity of both ventricles, leading to a heart condition [16]
Premature atrial contraction	PAC	Abnormal heart rhythm characterized by early depolarizations originating in the atria, the upper chambers of the heart [17]
Premature ventricular contraction	PVC	Abnormal heart rhythms occur when an early electrical impulse originates from the ventricles, the lower chambers of the heart [18]
ST-segment depression	STD	Specific abnormality observed on an electrocardiogram (ECG) where the ST segment, a portion of the ECG waveform, is lower in amplitude or depressed below the baseline [19]
ST-segment elevated	STE	Abnormality observed on an electrocardiogram (ECG) where the ST segment, a portion of the ECG waveform, is elevated above the baseline [20]

**Fig. 2:**
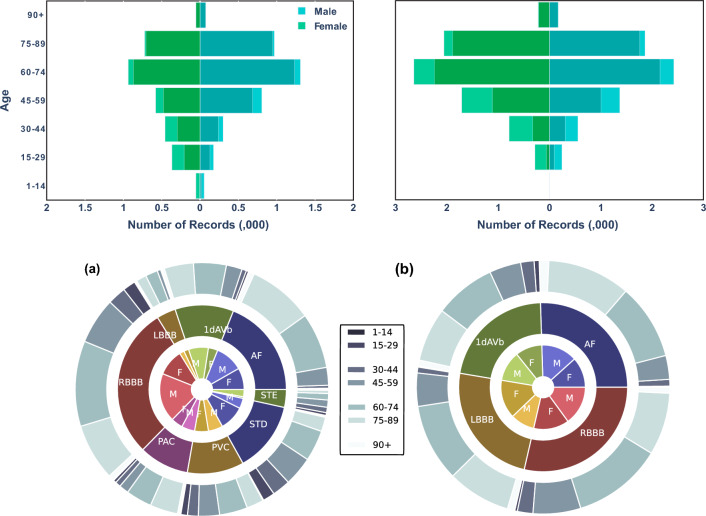
For **a**, the top left chart of the CPSC dataset displays age and gender categorization, where a darker bar represents the data related to abnormality. In the bottom left chart, the central doughnut graph presents the prevalence of various types of abnormality. The inner layer of the doughnut illustrates the distribution by gender, while the outer layer represents the distribution by age group. For **b**, the top right chart of the TNMG subset displays age and gender categorization, where a darker bar represents the data related to abnormality. The central doughnut graph in the bottom right chart presents the prevalence of various types of abnormality. The inner layer of the doughnut illustrates the distribution by gender, while the outer layer represents the distribution by age group

In the selection process, any data with only empty sample entries were excluded from the dataset. Consequently, the dataset comprises 6877 unique tracings. The data were reformatted into 4096 instances, and any extras were omitted from the dataset. The details of the refined dataset are outlined in Fig. 2a.

The figure reveals more male than female patients, indicating a gender imbalance in the dataset. Additionally, most individuals in the dataset are patients rather than non-patients. The age distribution of the patients follows a similar trend to that of the general population, with a larger proportion of individuals in the older age groups. However, there is a slight imbalance when examining the distribution of abnormality. The instances of LBBB and STE are relatively fewer than the other types of abnormalities in the dataset.

### TNMG

The TNMG dataset comprises 2,322,513 labelled 12-lead electrocardiogram (ECG) data featuring six distinct types of abnormalities [11], sampled at a rate of 400 Hz. To ensure conformity with the CPSC dataset, the data is resampled to 500 Hz. Of the six abnormalities present, four types overlap with those in the CPSC dataset, namely atrial fibrillation (AF), first-degree atrioventricular block (1dAVb), left bundle branch block (LBBB), and right bundle branch block (RBBB). The remaining two abnormalities, sinus bradycardia (SB) and sinus tachycardia (ST), do not overlap with the CPSC dataset. To evaluate the generalization performance of the trained model in a balanced dataset, 3000 samples were randomly selected from each of the four overlapping abnormalities, with an additional 3000 samples featuring no abnormality [21]. Consequently, the expected size of the sampled dataset is 15,000. However, given that some of the samples exhibit two or more abnormalities, the total size of the dataset is 14,332. Figure 2b below illustrates the dataset’s characteristics.

The dataset samples are standardized to a fixed length of 4096 at 500 Hz, with any exceeding this length being excluded from the data. This standardization process ensures consistency in the data’s structure, facilitating its analysis and modeling. The resampled dataset exhibits a balanced gender distribution, with a roughly equal proportion of males and females. The age distribution in the resampled dataset also follows the general trend of the population, with a higher proportion of individuals in the older age range. Additionally, the sampling process employed in creating the resampled dataset has resulted in a balanced distribution of the different abnormalities.

## Methods

We aim to develop a simple model that efficiently handles raw data and employs a shallow architecture suitable for implementation on hardware devices. Even though our tests had been conducted on 12-lead ECG, the low complexity architecture and lack of need for pre-processing features mean that the solution could enable simpler front-end electronics (e.g. reduced need for filtering) and highly accurate and portable heart monitoring system relying on a smaller number of leads in future. This includes hardware devices with limited processing capabilities, such as embedded systems, internet of devices or things, or edge computing platforms. It ensures optimal performance while utilizing the available hardware resources effectively. The model prioritizes simplicity and computational efficiency, enabling the direct processing of raw data without extensive filtering or feature engineering. This approach reduces latency and facilitates faster analysis of real-time data.

### Diagonal State Space Model

The model integrated Diagonal State Space Models, a widely recognized framework [4], renowned for its effectiveness in capturing and understanding the dynamics and interactions within time-series data. By incorporating the principles of Diagonal State Space Models into its design, the model demonstrated a dedicated focus on processing time sequence data. Diagonal State Space Models provided a robust framework for modelling and analyzing time-series data. They allowed for the representation of both observed and latent variables, which are unobserved variables that influence the behaviour of the observed variables. Using convolutional operations of S4D on signals instead of traditional convolutions on images empowered the model to effectively capture the underlying dynamics, dependencies, and interactions inherent within the data.

**Fig. 3:**
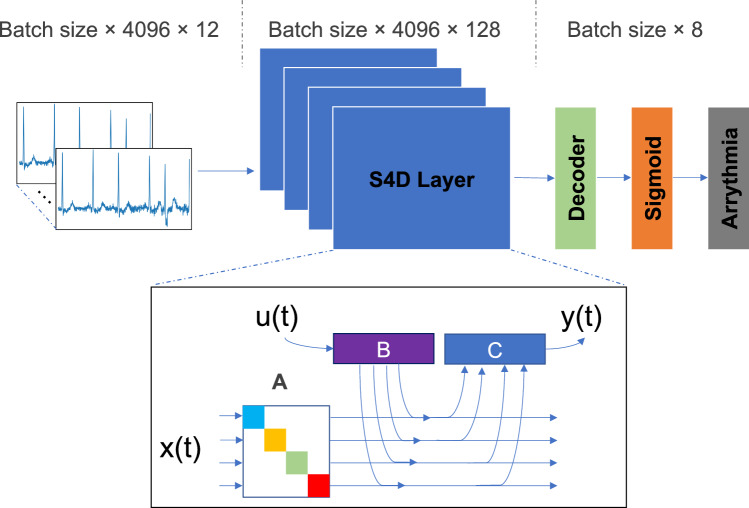
Proposed simple S4D model with raw input data, stacked S4D layers, and a decoder at the end, utilizing the sigmoid function to convert the output into abnormality types. In the S4D layers, the input signal *u*(*t*) is mapped to the output *y*(*t*) through a latent state *x*(*t*) using matrices *A*, *B*, and *C*

As shown in Fig. 3, the proposed model architecture was evaluated through experiments to determine the optimal number of S4D layers for achieving the best results. In contrast, a simple dense layer was employed as the model’s decoder to streamline the architecture.

### Evaluation Metrics

The performance metrics used in this paper are: Accuracy 5: The proportion of correct predictions out of the total number of predictions [22].

5 $$\begin{aligned} Accuracy = \frac{TP + TN}{TP + TN + FP + FN} \end{aligned}$$

F1-score 8: The harmonic mean of precision 7 and recall 6, which balances the trade-off between precision and recall.

6 $$\begin{aligned} Recall= & {} \frac{TP}{TP+FN} \end{aligned}$$

7 $$\begin{aligned} Precision= & {} \frac{TP}{TP+FP} \end{aligned}$$

8 $$\begin{aligned} F_{1}= & {} 2 \times \frac{Precision\times Recall}{Precision + Recall} \end{aligned}$$

The area under the Receiver Operating Characteristic Curve (AUROC): A measure of the model’s ability to distinguish between positive and negative cases at different threshold values. The Area Under the Precision-Recall Curve (AUPRC) is a performance metric for binary classification models. It quantifies the overall quality of the model by measuring the area under the precision-recall curve. A higher AUPRC value indicates better model performance, with 1.0 representing a perfect classifier. These metrics are commonly used in evaluating the performance of machine learning models for binary classification tasks such as abnormality detection.

## Experiment

### Training

We conducted experiments with different layer configurations to determine the optimal number of layers for S4D. We tested S4D models with 2, 4, 6, 8, and 10 layers while keeping other parameters constant. The key parameters used in these experiments included a learning rate of 0.001, 200 epochs, a batch size of 32, and a binary cross-entropy loss function. The CPSC dataset was employed to train the models, and the objective was to observe the variations in behaviour resulting from different architectural designs based on the number of layers used in S4D.

### Model Explainability

In addition to the previously mentioned hyperparameters used during model training, there is a crucial parameter called the ’model dimension,’ set to 128 in this case. This parameter controls the number of output channels produced by the S4D layer, and each output channel shares a similar structure to the input channels. This concept is analogous to performing convolutions on time-series data, making the S4D model particularly effective for processing time-series data like electrocardiograms (ECG). The core of the model’s power lies in the diagonal spectrogram space modulation kernel, which plays a pivotal role in transforming the convolutional ECG data. This kernel involves intricate calculations, including frequency domain transformations. In the S4D model, this kernel is computed based on the input data length, *L*. Subsequently, fast Fourier transforms (FFT) are applied to both the kernel and the ECG data in the frequency domain, effectively simulating a convolution-like operation. The result is then converted back to the original domain using an inverse FFT. The output undergoes further processing, including activation functions and point-wise convolutions. This comprehensive process empowers the S4D module to transform input data through intricate mathematical operations, ultimately extracting valuable features for use in deep learning models, making it particularly adept at handling time-series data like ECG. [4]

Incorporated into our training methodology is using a cosine annealing learning rate scheduler in conjunction with the AdamW optimizer. This particular scheduler orchestrates the modulation of the learning rate throughout the training process, simulating the behavior of a cosine wave. Its role in our training regimen is paramount, as it dynamically adapts the learning rate in tandem with the model’s learning progress. This adaptive modulation obviates the necessity for the conventional practice of early stopping to control the training process. Allowing the model’s learning rate to evolve organically ensures a more efficient and effective convergence in our training endeavors.

### In-Sample Test

A subset of 500 data points was randomly chosen from the CPSC dataset to test the model’s performance. These data points were not utilized during the model’s training phase but were exclusively reserved for evaluating the model’s performance. Using this independent test dataset, we can assess how well the model generalizes to new, unseen data and obtain a more accurate understanding of its overall performance. The best-performing model is selected based on the performance results obtained from the testing phase. The selection process involves comparing the performance metrics of different models and choosing the one that demonstrates the highest level of performance.

### Out-of-Sample Test

The selected model was rigorously tested on the TMNG dataset, consisting of 14,332 ECG data points. This dataset is specifically chosen to evaluate the model’s performance in real-world scenarios. An out-of-sample test will assess the model’s ability to generalize and accurately predict unseen data. The predictions will be compared to known values to measure performance metrics. The results will be recorded and analyzed to understand the model’s strengths and limitations. This evaluation will inform decisions regarding the model’s effectiveness and potential refinements for improved ECG analysis.

### Robustness Test

During the robustness testing phase, the model will undergo evaluation by randomly emptying leads from the 12-lead ECG data. The aim is to assess the model’s ability to maintain performance even when specific leads are missing. The test will be conducted by progressively emptying different numbers of channels, specifically 2, 4, 6, 8, and 10 leads. Performance metrics will be used to evaluate the model’s robustness in handling missing leads. By comparing the model’s performance across the different scenarios, we can gain insights into its ability to maintain accurate predictions and interpret ECG data effectively, even when specific leads are absent. The robustness test results will be carefully evaluated to determine how well the model handles the missing lead scenarios. This assessment will help identify potential limitations or areas for improvement in the model’s robustness. By understanding the model’s performance under these conditions, we can make informed decisions about its suitability for practical applications where missing leads may occur in real-world ECG data.

### Training with Single-Lead ECG

As an integral component of our overarching study, we conducted a specific investigation to evaluate the model’s performance in the context of single-lead ECG (Lead II). The model underwent exclusive training using the Lead II data from the dataset. Subsequently, we compared its performance when exclusively exposed to single-lead ECG against the model trained on the complete 12-lead ECG dataset. It is important to note that the training process and model architecture remain identical to the 12-lead examination. The sole difference lies in using Lead II ECG data for training and testing purposes, offering a focused understanding of the S4D model’s capabilities in this specific context.

## Result

The results of the experiment are delineated in the ensuing two subsections. The initial subsection showcases the discoveries of the proposed model, whereas the subsequent subsection scrutinizes the proficiency of the proposed model in the context of generalization.

### Pre-processing of Data

The results obtained from both denoised and non-denoised data, as demonstrated in Table 2, indicate that the proposed model can handle noise effectively for abnormality identification. The denoising techniques employed in the study included a 4th order bandpass filter with a low-cut frequency of 0.5 and a high-cut frequency of 40. Additionally, wavelet denoising was utilized with the ‘db4’ wavelet, a decomposition level of 8, a cutoff frequency of 0.1 Hz, and a 6th order filter. These findings suggest that the step of data pre-processing may not be necessary, as the model performs well even without extensive pre-processing techniques.

**Table 2:** Model performance on denoised & raw data

Data	F1 (%)	AUROC (%)	AUPRC (%)
Pre-processed	84	98	91
Raw	85	97	91

To evaluate the burden associated with ECG pre-processing, we conducted an experiment involving the pre-processing of a dataset comprising 14,332 ECG data points from the TNMG subset. This pre-processing task, which included the application of bandpass filters and denoising techniques to the ECG data, consumed approximately 3 min of computational time. The experiments were performed on an Intel Xeon CPU E5-2630 v3 (at 2.4 GHz). It is important to highlight that the latency would likely be even more pronounced if such pre-processing were carried out on a smaller edge device. Consequently, the model’s capability to operate efficiently without needing extensive pre-processing emerges as a valuable feature.

### In-Sample Training and Validation

**Fig. 4:**
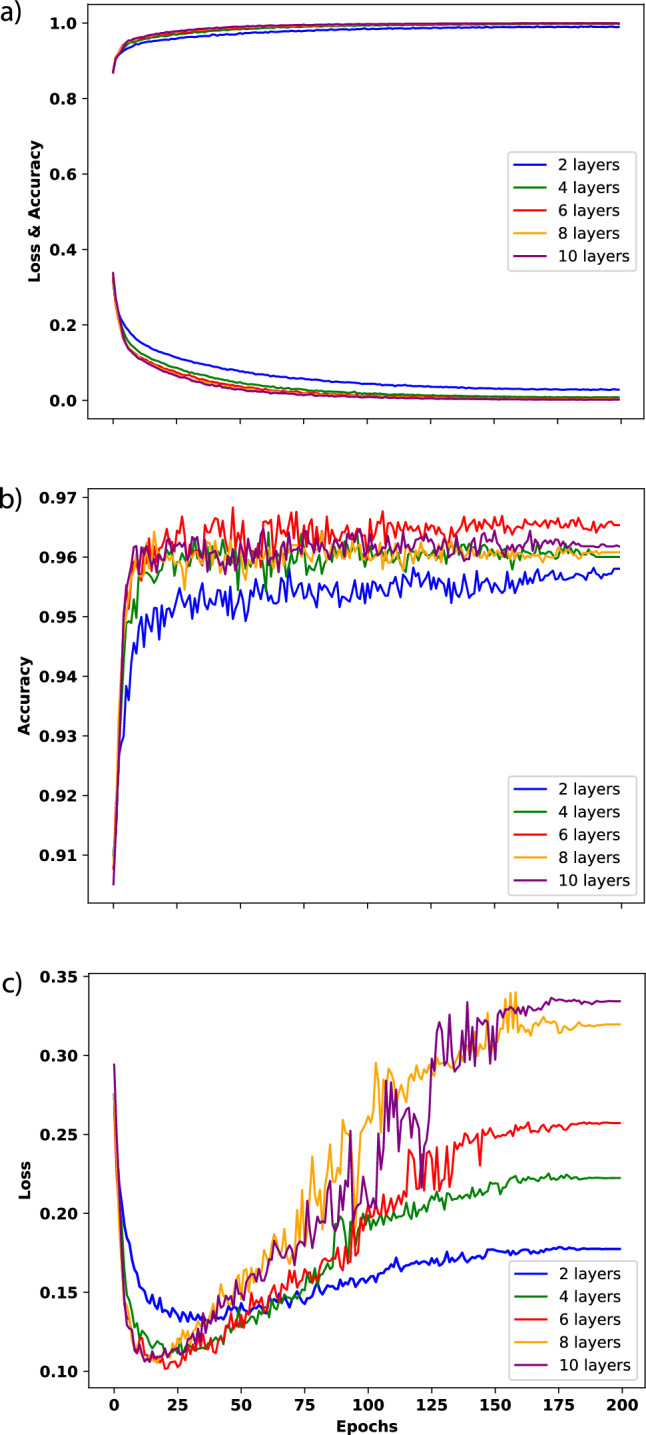
Recorded loss and accuracy metrics. **a** Illustrates the loss and accuracy metrics performance during the training process of the S4D model with varying layers. **b** Shows the accuracy metric performance during the validation process of the S4D model with varying layers. **c** Demonstrates the loss metric performance during the validation process of the S4D model with varying layers

This section will provide insights into the in-sample observations derived from the model training and validation results on the CPSC dataset. The present study trained the proposed model on the previously introduced CPSC dataset with 200 epochs. Throughout the training process, both the training and validation loss and accuracy were recorded per epoch. Figure 4 visually represents the recorded loss and accuracy metrics for the training and validation sets during the entire training procedure. Furthermore, the S4D model underwent various modifications with different layers, and the obtained results are concisely summarized.

As depicted in Fig. 4a, the training loss steadily declined throughout the training process while the training accuracy increased and stabilized. While higher layer configurations in the S4D model generally yield improved performance during training, the corresponding increase in accuracy is not significant beyond four layers. Our observations indicate that further increasing the number of layers can result in a moderate improvement in the model’s performance, although the extent of this improvement is not particularly substantial. Consequently, in light of our study’s findings, we have chosen to utilize a four-layer model.

Throughout the training process, the validation accuracy generally displayed an increasing trend. However, notable fluctuations could also be observed until approximately 175 epochs, after which the accuracy stabilized. Additionally, Fig. 4b reveals that, apart from the simplest 2-layer model, the number of layers in the S4D model did not significantly affect the validation accuracy. Figure 4c depicted the validation loss metrics during training. It could be observed that the validation loss exhibited a general trend of initially decreasing and then increasing, with notable fluctuations until approximately 175 epochs, after which it stabilized. Moreover, it was evident that, for the S4D model, the number of layers directly influenced the final validation loss, with higher layer configurations resulting in higher stabilised losses.

After analyzing the behaviour of various models during the training process, a four-layer model was chosen as the foundation for further analysis. This model exhibited desirable characteristics, including high accuracy and stability in training and validation performance. Furthermore, the four-layer model offered a simpler architecture than other alternatives, as it has a shallow structure. This decision was made based on the model’s performance and the desire for a more straightforward and interpretable model design.

**Table 3:** Model performance on the CPSC test-set

Class	F1 (%)	AUROC (%)	AUPRC (%)	Accuracy (%)
1dAVb	85.2	96.2	92.9	96.8
AF	92.0	98.2	95.8	97.2
LBBB	96.8	100.0	100.0	99.8
RBBB	91.0	98.8	96.9	95.0
PAC	57.5	92.0	61.1	93.8
PVC	74.4	92.6	76.3	95.6
STD	83.8	97.7	91.3	95.2
STE	66.7	97.8	65.4	98.4
Weighted average	84.4	97.6	91.1	96.5

According to Table 3, the 4-layer model effectively detected abnormality, with a weighted average F1-score of 0.844 and an AUROC of 0.976. Notably, the model demonstrated strong performance in abnormality identifications.

Additionally, various AI models have emerged as potential tools for diagnosing abnormalities in the current landscape. Since the F1 score serves as the universally adopted performance metric across the majority of studies utilizing the same dataset (as indicated in Table 4), it is evident that our model consistently outperforms or matches the performance of existing approaches, including the Combination of Deep Residual Network and Bidirectional LSTM (ResNet & BiLSTM), spatiotemporal attention-based convolutional recurrent neural network (ST-ACRNN).

**Table 4:** Comparison of model performance

	Model type	F1 (%)
[23]	ResNet & BiLSTM	80.6
[24]	ST-ACRNN	83.5
[2]	CNN	84.5
Our work	S4D-CfC	84.4

### Model Generalisation

In order to evaluate the generalization performance of the selected model, we utilized the models trained on CPSC to make predictions on the TNMG subset data, which was previously introduced in the data section. However, the original TNMG dataset consists of six distinct types of abnormalities and only four overlap with the CPSC dataset. To ensure a balanced dataset, the TNMG subset was specifically created for this purpose. By testing the chosen model on this subset, we can gain insights into its ability to generalize beyond the training data.

**Table 5:** Model generalisation performance on TNMG subset

Class	F1 (%)	AUROC (%)	AUPRC (%)	Accuracy (%)
1dAVb	74.8	93.0	83.4	90.9
AF	71.1	94.4	87.6	88.9
LBBB	90.6	97.9	95.8	95.9
RBBB	87.2	96.4	92.3	92.9
Weighted average	81.2	95.5	89.6	92.2

Table 5 demonstrate that the model generalizes well, achieving an average F1-score of 0.812 and AUROC of 0.955. This finding illustrates that with a balanced dataset, the model’s generalization capability can achieve high levels of accuracy.

### Model Robustness

Based on the promising generalization results achieved with the model, we further evaluated the model on the TNMG dataset under conditions where some channels of the 12-lead ECG data were randomly emptied.

**Fig. 5:**
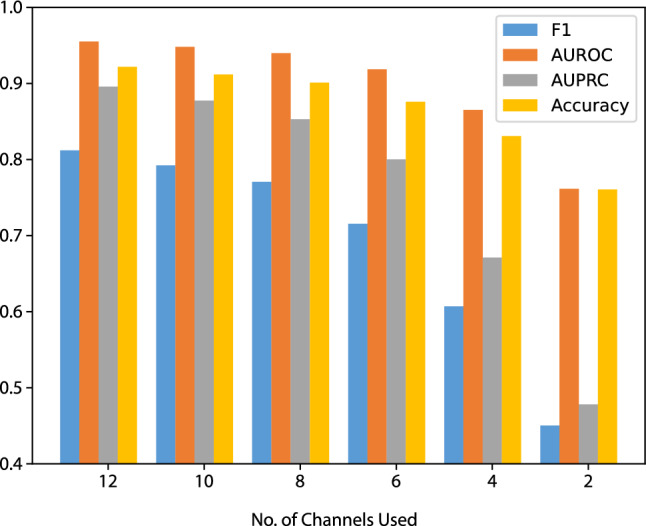
The model’s performance is evaluated with varying number of leads from the 12-lead ECG data during inference. The vertical axis represents the weighted average metric scores

As shown in Fig. 5, the model’s performance decreases as the number of emptied leads increases. However, the model remains highly robust even with half of the 12-lead emptied, with an F1-score above 0.7. These results suggest that the model can handle missing channel data and maintain its accuracy, indicating its strong robustness.

### Evaluation Using a Single-Lead ECG

As a component of our inquiry into the model’s performance, we executed an experiment utilizing the CPSC dataset. Remarkably, in this particular experiment, we exclusively employed Lead II data for training the model. The ensuing results are meticulously detailed in Table 6.

**Table 6:** Performance of the model with single-lead ECG data

Class	F1 (%)	AUROC (%)	AUPRC (%)	Accuracy (%)
1dAVb	82.8	97.7	91.1	96.0
AF	93.2	98.2	96.2	97.6
LBBB	88.2	99.9	96.2	99.2
RBBB	81.0	92.8	89.0	90.4
PAC	49.4	86.8	42.0	91.4
PVC	69.0	93.5	78.1	94.6
STD	82.6	97.0	89.0	94.6
STE	54.5	96.0	54.4	98.0
Weighted average	79.5	95.7	86.4	95.2

Comparing the results in Table 3, which were obtained from a model trained on 12-lead ECG data, the performance of the model trained solely on Lead II data exhibits intriguing characteristics. While there is a slight marginal decrease in the model’s overall performance across eight abnormalities, it is noteworthy that this model was trained using data from just a single lead. Despite this constraint, the model’s performance remains quite commendable.

## Discussion

The experiment results indicate that the proposed model for abnormality identification is effective and robust in handling noise. The model demonstrated good performance on both denoised and raw data. The lack of need for a pre-processing or feature extraction step means the system is simpler, architecturally less complex and capable of being deployed in resource-constrained applications such as for portable daily use.

The four-layer model effectively detected abnormalities on the CPSC test set, achieving a weighted average F1-score of 0.844 and an AUROC of 0.976. These results indicate that the model can accurately identify various types of abnormality. Notably, the model achieved solid performance in detecting LBBB, with outstanding F1-score, AUROC, and AUPRC. These highlight the model’s ability to classify specific abnormality classes accurately.

To assess the generalization capability of the selected model, it was tested (inference only) on an out-of-distribution independent dataset, the TNMG dataset from Brazil. The model exhibited good generalization performance with an average F1-score of 0.812 and an AUROC of 0.955. These findings indicate that the model can effectively generalize beyond the training data and accurately classify abnormalities in previously unseen datasets, which is not a common practice observed in many of the publications in this domain and can reliably constitute a pseudo-perspective method of testing and predicting algorithms’ clinical performance.

Furthermore, the model’s robustness was verified by randomly emptying channels of the 12-lead ECG data in the TNMG dataset. The results from this test showed that even with half of the leads emptied, the model remained highly robust and kept an F1 score above 0.7. This demonstrates the model’s ability to handle missing channels, a real-world scenario where a complete 12-lead ECG may experience a sudden or persistent lack of data from one or more leads.

Overall, the proposed model exhibits strong performance in abnormality identification, with good generalization capability and robustness to missing channel data. The model’s simplicity, interpretability, and high accuracy make it a promising tool for abnormality detection in clinical settings. Future research is under development, focusing on evaluating the model’s performance on more extensive and diverse datasets relative to cardiologist performance to be used as an AI-assistant tool in annotating or interpreting ECG data. We hope that the model can be part of a more significant deployment to portable devices and reduces the resources required (memory and energy needs) for near-sensor data analysis.

## Study Limitations

*Limited Abnormality Classes* One notable limitation of this study is the focus on a specific set of cardiac abnormalities in the CPSC dataset. While the model’s performance in identifying these particular abnormalities has been assessed and found promising, it’s important to recognize that its generalization to other, less common cardiac conditions or abnormalities not covered in the dataset remains unexplored. Therefore, the model’s applicability and accuracy for a broader spectrum of cardiac anomalies may be limited and require further investigation and validation using diverse datasets encompassing a wider range of cardiac pathologies.

*Data Source Dependency* This study heavily relies on the CPSC and TNMG datasets for training, validation, and testing. These datasets, while comprehensive, may not fully represent the diversity of real-world clinical scenarios and patient demographics. As a result, the model’s performance may be tailored to the characteristics and biases present in these datasets. To enhance the robustness and real-world applicability of the model, future research should consider incorporating data from multiple sources and populations, ensuring a more comprehensive evaluation of its performance across different clinical settings and patient groups.

*Dataset Bias* This study relies on the availability and quality of the CPSC and TNMG datasets for training and evaluation. It’s important to acknowledge that these datasets may have inherent biases due to data collection procedures, patient demographics, and data sources. Therefore, the model’s performance may be influenced by the characteristics of these datasets, potentially limiting its generalizability to more diverse or representative populations. Efforts to mitigate dataset bias and explore the model’s performance on broader and more diverse datasets could provide valuable insights for future research.

*Lack of Real-Time Evaluation* The evaluation of the model’s performance in this study is based on batch processing of ECG data. However, real-time evaluation of the model’s capabilities is not addressed, particularly in a clinical setting where immediate decisions may be critical. Incorporating real-time evaluation and assessing the model’s response time in a clinical context could be an important avenue for future research to ensure its practical utility in time-sensitive scenarios. As we advance towards more sophisticated hardware implementations, including edge devices focusing on off-the-shelves microcontrollers, microprocessors, and custom integrated circuits, current and future studies are underway to address these limitations.

## Conclusion

This research article introduces a demonstrated algorithm that utilizes the S4 model to process raw and long sequences of ECG data. The algorithm exhibits robustness against noise and demonstrates high efficacy. It is trained on the CPSC dataset (from China) and generalized (inference-only) on the TNMG dataset (from Brazil) subset to test its generalization capabilities. The results showcase an impressive average F1-score of 0.812, providing substantial evidence of the algorithm’s robustness in classification. Furthermore, the AUROC metric achieves a score of 0.955, indicating the model’s notable discriminative ability.

One noteworthy advantage of this algorithm is its ability to handle noise without needing a pre-processing or feature extraction step. We have demonstrated that the accuracy of the results remains unchanged, with or without these steps, affirming the algorithm’s capability to handle noise effectively. As a result, the algorithm offers a low-complexity architecture, enabling deployment on various devices such as embedded systems, internet of medical devices, or near-sensor computing platforms for hospitals and home use. This versatility allows the algorithm to perform even in resource-constrained environments (i.e. hardware), making it a valuable solution for a wide range of real-world applications.

## Data Availability

This paper employs a publicly accessible CPSC dataset and the TNMG dataset, which can be obtained by requesting access from the data owner. The dataset is not publicly available, but access can be granted upon request with approval from the custodian of the data.

## Code Availability

The code is available on GitHub at https://github.com/NeuroSyd/S4D-ECG. The code is built upon the foundation laid out in [25].
